# Clinical outcomes after carbon-plate osteosynthesis in patients with distal radius fractures

**DOI:** 10.1186/s13037-019-0210-8

**Published:** 2019-09-04

**Authors:** Florin Allemann, Sascha Halvachizadeh, Thomas Rauer, Hans-Christoph Pape

**Affiliations:** Department of Traumatology, University of Zurich, University Hospital Zurich, Raemistrasse 100, 8091 Zürich, Switzerland

**Keywords:** Carbon implant, CF/PEEK plates, Plate osteosynthesis, Wrist fracture, Distal radius fracture

## Abstract

**Background:**

Surgical implant material has changed over time, from metal to stainless steel to titanium. In recent decades a new material, carbon-fibre-reinforced polyether ether ketone, has been introduced. The aim of this study was to assess the clinical and radiological feasibility and functional outcome after treatment of distal radius fractures with this new implant.

**Methods:**

Inclusion criteria: AO type B distal radius fractures treated with 2.7 mm CF/PEEK plates at one Level 1 trauma centre between 2016 and 2017. Follow-up period 1 year, measurement of range of motion and radiographic assessment, histological analysis of debris only after plate removal.

**Results:**

Out of 112 eligible patients, 10 (8.9%) patients were included. Mean operation time was 65 ± 10 min. Radiographic healing was confirmed by radiologists at 6 weeks follow-up. During one-year follow-up, no adverse events were reported and functionality and patients subjective satisfaction improved significantly (*p* < 0.05). Only one plate was removed, with no histological signs of inflammation or allergic reaction.

**Conclusions:**

The 2.7 mm CF/PEEK plate osteosynthesis appears to be a reliable and safe implant for certain types of distal radius fracture. Assessment of fracture union is substantially more practical and functionality improved significantly over 1 year.

## Background

Implant biocompatibility is a major safety issue in orthopaedic fracture care. Historically, there have been issues with materials that do not consist of certain metals or metal combinations [1]. Incompatibility may occur with a time delay after implantation. Specifically, early carbon implants for anterior cruciate ligament (ACL) replacements have led to issues of failure and local tissue irritation. Likewise, non-metal implants have caused local inflammatory reactions that precluded them from further clinical use [2, 3]. Along with the improvements in material development, new implant combinations have been developed that appear to be safe for routine clinical use. In this way, polyether ether ketone (PEEK) has been added to carbon [4–6]. In previous clinical applications, CF/PEEK implants have been used successfully to cover osseous defects in neurosurgical patients [7, 8]. They have been favoured due to their abilities to sustain plastic deformation and their ability to mimic anatomic structures in other fractures as well, e,g., of the distal radius [9]. Meanwhile, various implants have been developed for orthopaedic fracture care, such as intramedullary nails, angular stable plates [10], and bar-to-bar constructs for spinal surgery. However, due to the relative novelty of the implant material, there are only a limited number of articles investigating clinical and functional outcomes of carbon-plate osteosynthesis. Therefore, the aim of this study was three-fold:

1. To assess the clinical application of plate osteosynthesis using 2.7 mm CF/PEEK plates in acute distal radius fractures,
2. To analyse the radiographic outcome and fracture consolidation of distal radius fractures treated with 2.7 mm CF/PEEK implants, and
3. To compare the development of functional outcome during routine clinical follow-up.

## Methods

### Ethical considerations

This study follows the guidelines of Helsinki [11] and was conducted after the approval by the local Zürich ethics committee (Kantonale Ethikkommission Zürich, KEK number 2018–00146).

### Study population and design

This retrospective cross-sectional cohort study included adult patients treated due to a distal radius fracture at one Level 1 trauma centre between 2016 and 2017. Inclusion criteria included: displaced distal radius fracture in need of surgical stabilisation; AO type B fractures; and complete data at one-year follow-up period. Exclusion criteria included: AO type C fractures; carpal instability requiring additional implants (screws or plates); open fractures; patients with genetic disorders affecting the musculo-skeletal system; oncology patients; and multiply injured patients. All patients were surgically treated by one surgeon (FA).

### Implant

All patients were treated with a 2.7 mm CF/PEEK plate (Inc. Icotec, Altstätten, Switzerland). Its design is oriented towards the conventional 2.4 mm two-column titanium plates (De Puy/Synthes, West Chester, PA). The implant follows the principles of the three-column theory [12] and allows for monoaxial screw placement.

### Follow-up

During the one-year follow-up period, patients received routine check-ups at 6 weeks, 12 weeks, and 1 year after surgery. The follow-up was performed exclusively by the treating surgeon. The follow-up included routine clinical examination, including range of motion (ROM), pain on exertion, neurological examination, subjective comfort of the patient, and routine radiographic examination. Any issue of biocompatibility or discomfort possibly related to the implant was addressed specifically.

### Implant removal and histology

Implant removal was performed only if medically necessary (infection, neuro-muscular damage) or after the patient’s explicit wish. During implant removal biopsies of the granulation tissue at the screw holes of the plate were taken for histological analysis. Histological analysis included haematoxylin and eosin (HE) staining, cell count for inflammatory cells, and allergic responsive cells. Further, the histological probes were analysed for carbon debris and possible cellular incorporation of carbon debris.

### Outcome measures

The primary outcome was improvement in ROM 1 year after surgery. This was assessed during routine clinical follow-up visits and measured in degrees (°). Further outcome parameters were radiographic union and the safety and feasibility of the use of CF/PEEK implant for surgical treatment of the distal radius fracture.

### Statistics

Continuous variables are displayed as means with standard deviation, and categorical variables as numbers and percentages. The progress of ROM was compared by Students t-test and corrected for multiple testing. Categorical variables were compared using the chi-square test. Figures were designed using GraphPad Prism version 8.1.2. for Windows (GraphPad Software, San Diego, CA, USA; www.graphpad.com). Statistical tests were preformed using R (R Foundation for Statistical Computing, Vienna, Austria. www.R-project.org).

## Results

Out of 112 distal radius fractures, ten patients (8.9%) were eligible and consented to be treated with a CF/PEEK implant. All included patients completed the follow-up period. These patients were 53.3 ± 16.6 years old and the male/female ratio was 60:40.

### Perioperative course

The mean time of surgery was 65 ± 10 min. During surgery, two screw head breakages occurred in two different patients. Those holes were left unmounted. No further surgery-associated complications were observed during the follow-up period. The CF/PEEK plates were placed correctly under the watershed line and all distal CF/PEEK screws were positioned without affecting the extensor tendon pockets. Of note, there was intraoperative breakage of a screw head in two screws (case nos. 2 and 5) and the screw holes were left unmounted. This did not lead to any complications.

### Postoperative course

There was no incidence of wound breakdown postoperatively. No secondary secretion or drainage was observed. All patients sustained the routine protocol for staged physical therapy and did not report any implant-associated issues at any time. There were no extensor tendon problems. Algodystrophy did not occur in any patient.

### Radiographic outcome

**Fig. 1:**
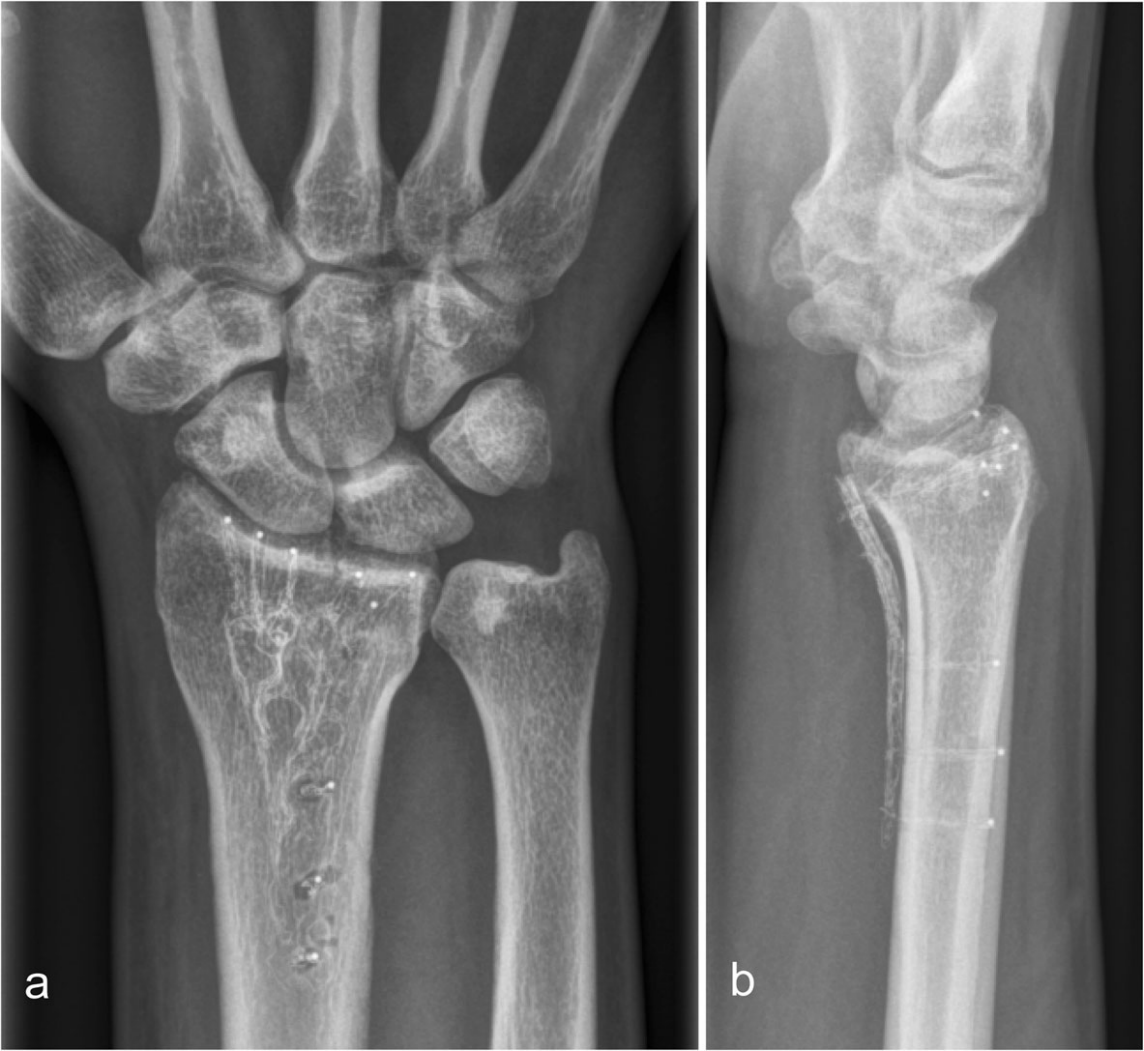
Distal radius fracture treated with a 2.7 mm Carbon/PEEK plate. X-ray 6 weeks after surgery anteroposterior (ap) (**a**) and lateral (lat) view (**b**)

Healing was uneventful and was completed at 6 weeks in all patients (Fig. 1).

**Fig. 2:**
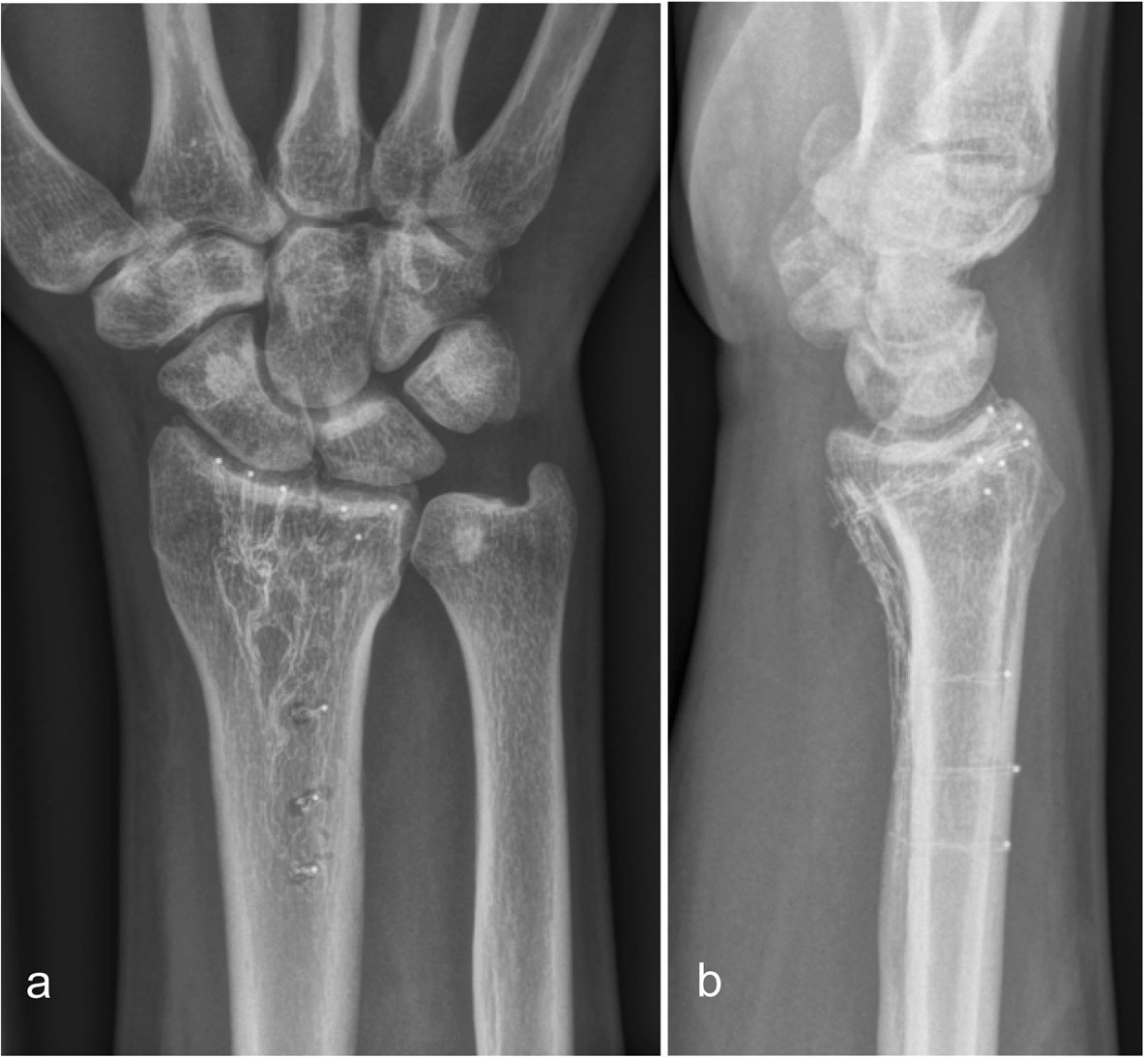
Distal radius fracture treated with a 2.7 mm Carbon/PEEK plate. X-ray 12 weeks after surgery, ap (**a**) and lat view (**b**)

**Fig. 3:**
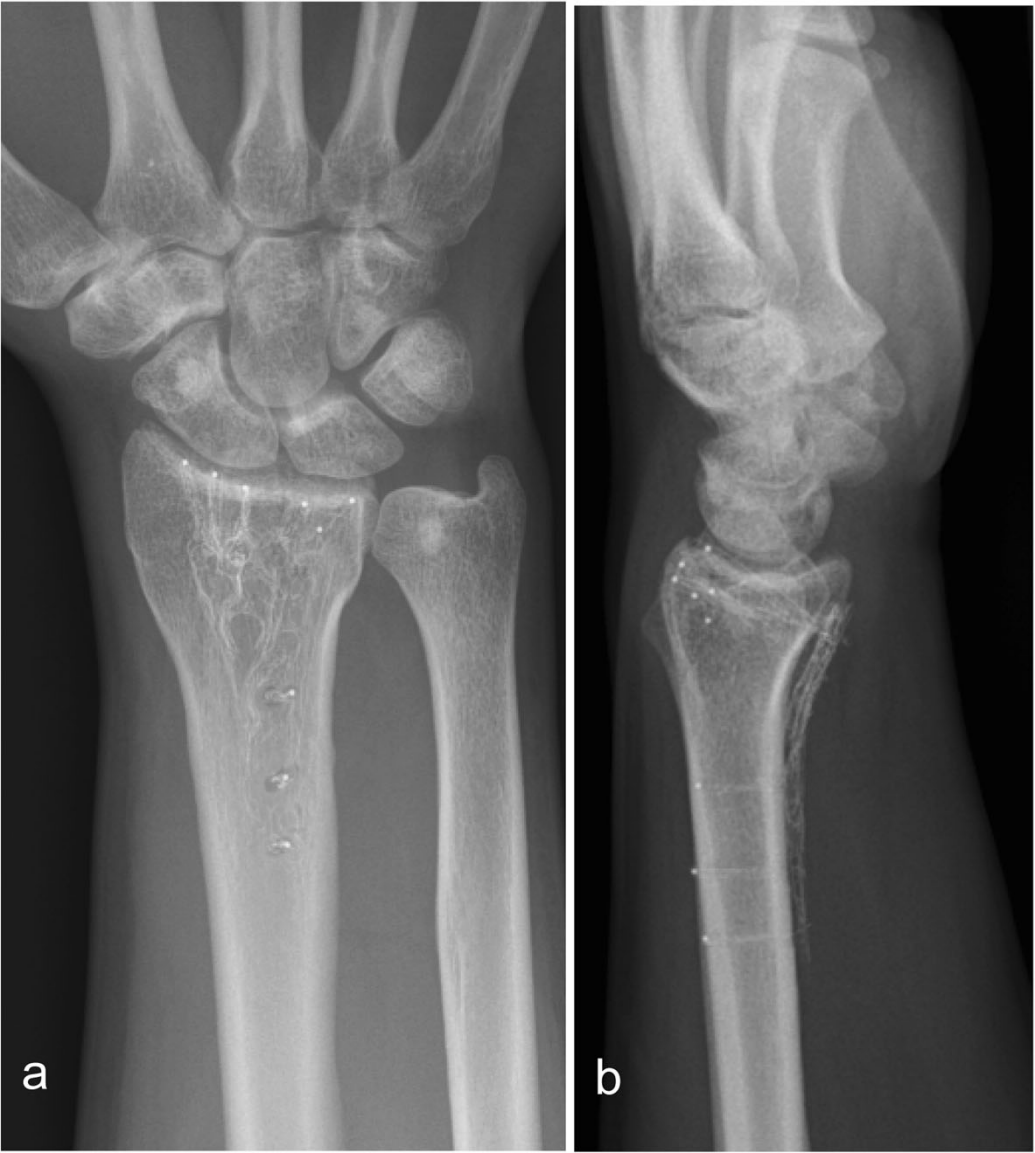
Distal radius fracture treated with a 2.7 mm Carbon/PEEK plate. X-ray 1 year follow up ap (**a**) and lat view (**b**)

At 12 weeks (Fig. 2) and at 1 year (Fig. 3) there was no loss of reduction. No implant failure, screw breakage or loosening was observed. The radiolucent properties of the implant facilitated substantially the assessment of union.

### Follow-up

**Fig. 4:**
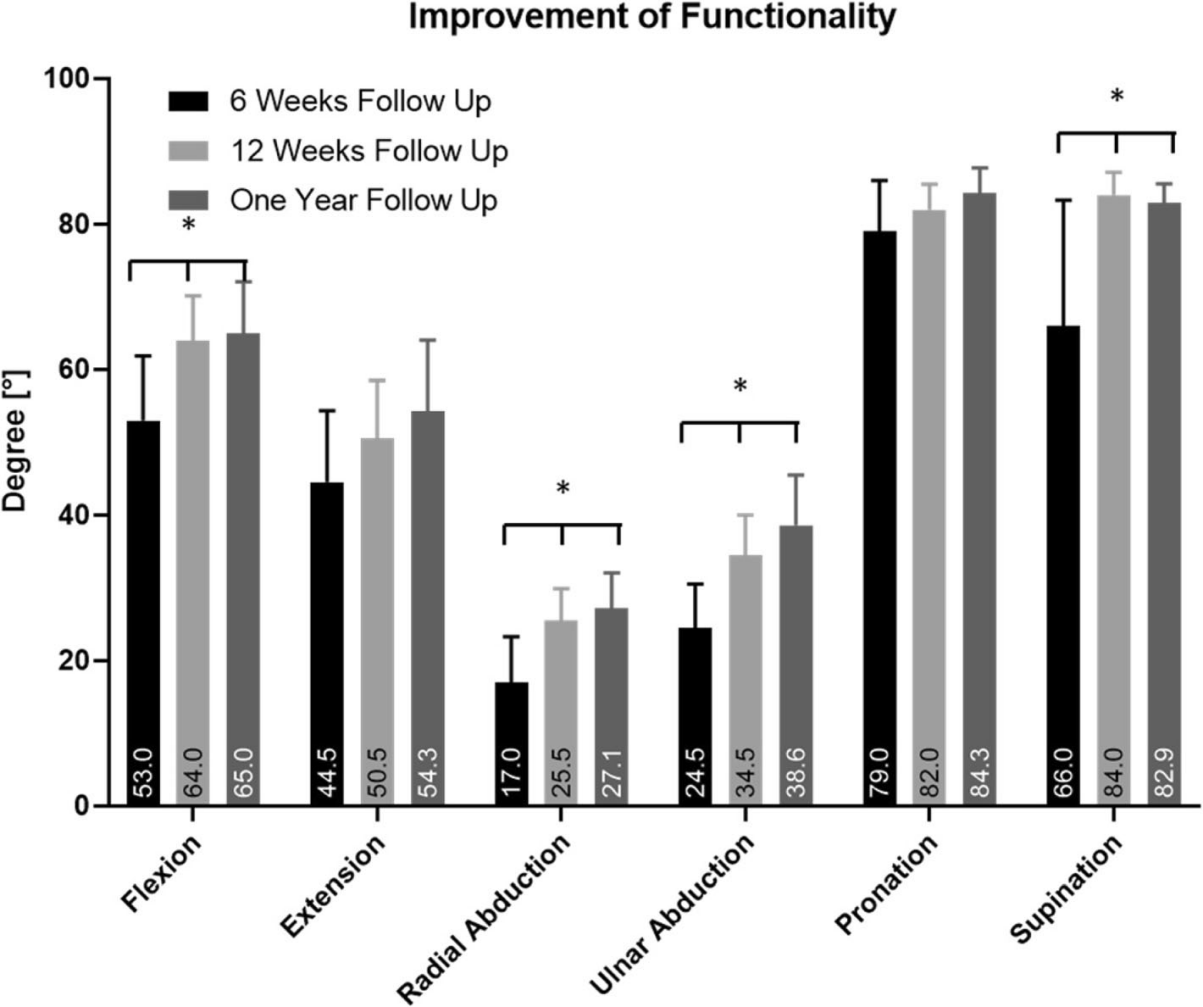
Improvement of Range of Motion during the Follow up after treatment with 2.7 mm. CF/PEEK distal radius plate. * = *p* < 0.05; statistical significant increase in range of motion during follow up period

Four male (age range: 32–66 years) and six female (age range: 45–78 years) patients were re-examined. There was no carpal tunnel syndrome at follow-up. Flexion increased significantly by a mean of 15 ± 13.0°, *p* = 0.0004. Extension increased by a mean of 26.7 ± 16.3°. Radial abduction and ulnar abduction increased significantly by a mean of 10 ± 8.4°, *p* = 0.003 and 5.8 ± 10.2°, *p* < 0.0001. Pronation increased by 12.5 ± 11.7° and supination significantly by a mean of 8.3 ± 5.2°, *p* = 0.004. These changes are shown in Fig. 4.

### Histological analysis

Histological analysis was performed in only one patient (10%). None of the patients had any medical reason for implant removal. Only one patient wished the implant to be removed, since she did not want any foreign implant in her body. At the time of implant removal (1 year after implantation), the fracture was healed. The gross inspection during implant removal did not show any signs of inflammation, hyper-perfusion, or swelling. HE staining of granulation tissue revealed no increased numbers of inflammatory cells, no increased numbers of eosinophils, and no implant debris. The pathologists did not describe any adverse histological reactions or allergic reactions.

## Discussion

CF/PEEK implants represent a new material for surgical treatment of fractures. Numerous studies have investigated preclinical properties and biomechanics of this material and showed advantages as well as disadvantages compared to other materials [13–15]. This study aimed to investigate the clinical application of CF/PEEK plates for distal radius fractures and concluded the following points:

1. CF/PEEK implants are a safe and reliable implant in the treatment of acute distal radius fractures (AO type B),
2. Radiographic assessment is easier due to the unique property of radiolucency of CF/PEEK implants, and
3. The functional outcome improves significantly 1 year after treatment with CF/PEEK implant.

Angular stable plating has revolutionised the treatment of distal radius fractures [16]. While loss of reduction has been a frequent problem in the era of conventional plating, this complication was reduced tremendously by the use of volar angular plates [10]. Monoaxial screw fixation is usually sufficient in these situations as long as proper fracture reduction is achieved. Numerous studies have shown that volar supply also showed a good functional outcome in complex intra-articular fractures [17]. This could be dispensed with the additional supply of dorsal of complex distal radius fracture, and the frequent tendon injuries due to the compromise of the tendon pockets could be avoided. A new area began with the treatment of distal radius fractures with titanium plates and angle-stable screws. However, these developments also involved some difficulties.

Above all, three problem areas can be identified: First, due to metal artefacts one may not be able to evaluate osseous consolidation in some cases. This may result in more conventional X-ray controls; Second, metal artefacts in the immediate vicinity of the TFCC can aggravate a violation of this complex in terms of diagnostics; Third, the nature of titanium plates is not similar to that of natural bone. Conventional titanium plates are more rigid than biological bone [18]. In addition, plate design and screw thickness play essential roles in rigidity [19]. Studies with CF/PEEK implants show that this material has similar strength and rigidity to the titanium plates used today [20]. CF/PEEK is even closer to the biological properties of the bone structure.

In our series, monoaxial screws appeared to be sufficient for fixation of the intra-articular fractures selected; namely, AO type B injuries with no additional ulnar instability.

Our study has certain limitations. Certainly, the sample size was limited and the overall follow-up was around 1 year. However, we feel that despite the limited number, our study is valuable for the following reasons: 1. It represents a single-surgeon experience for surgery and two experienced surgeons for follow-up. 2. In all patients, we waited to submit the clinical result after completion of physical functioning and return to work. In distal radius fractures, this is achievable within 3 months after injury. 3. We were pleased with the radiolucency of the implant, allowing for proper assessment of healing in every X-ray on follow-up. In this respect, radius fractures differ from other injuries, such as tibial or femur shaft fractures.

In general, the individual functional score results and the mobility of the wrist joint demonstrated favourable-to-excellent results in all cases, which are in accordance with other studies [21, 22]. Specifically, in the patients that had screw breakage intraoperatively, this did not result in clinical drawbacks. Since the screw breakage occurred in the first patients only, we feel that it represents the learning curve in dealing with a new type of implant. Regardless, in our series the general duration of surgery was comparable to that using conventional titanium implants. On re-examination, all patients were back to their regular activities and no limitations were observed.

During follow-up, no patient reported problem in regards to the selected implant; namely, skin irritation, wound healing, signs of soft tissue irritation, swelling or redness. None of the patients developed implant-associated pain, infection, allergic reactions, or syndromes of neurological irritation, such as those described in carpal tunnel syndrome. There were no other clinical symptoms associated with wear of the implant, or other sign of wear.

In our results, none of the patients had any issue of bone healing, and the radiological assessment allowed for timely recovery in all cases. This is in line with Di Maggio’s results [23]. The authors examined patients with CF/PEEK implants and pointed out that CF/PEEK allows for assessment with no radiological issues resulting from the artefacts [23]. These studies are in accordance with De Jong’s results [24].

### Strengths and limitations

As the management and treatment strategies of distal radius fractures might differ between institutions and even between treating physicians, our single-centre investigation decreased the chance of including a bias resulting from different treatment strategies. One might argue that the inclusion of type B fractures only might have improved the outcome; however, we feel that the concentration on one specific type entity of fracture reduced the heterogeneity of outcome measures. One limitation of this study was the sample size. It was not designed as a controlled cross-sectional study, and in order to be able to show non-inferiority of this implant compared to routinely used implants a higher sample size would be necessary. However, this study showed the clinical application with a reasonable follow-up period and might therefore serve as groundwork for future research to assess and compare different implants for the surgical treatment of fractures. The histological analysis of only one patient was not representative and should not be generalised. However, since none of the patients showed clinically relevant complications that led to implant removal, we feel that this implant may be safe to use in selected patients.

Further research is needed to assess long-term outcomes as well as comparisons of implant types with larger sample sizes.

## Conclusions

Our study showed the clinical and radiological application of CF/PEEK implants in distal radius fractures. We conclude that this implant is safe to use in patients with B-type fractures of the distal radius. Radiologic assessment of fracture union was improved and functional outcome improved significantly over 1 year.

## Data Availability

All data generated or analysed during this study are included in this published article. The datasets are not publicly available due to privacy limitations but are available from the corresponding author by reasonable request.

## Abbreviations

AO: Classification system of fractures
CF/PEEK: Carbon-fibre-reinforced polyether ether ketone
PEEK: Polyether ether ketone
